# Sawdust Urinals

**Published:** 1898-09-17

**Authors:** 


### SAWDUST URINALS.

Dr. Vivian Poore advocates the use of sawdust as an absorbent material in urinals, and it is a suggestion which might usefully be adopted in many cases, especially when as at present the water supply runs short. In the construction of such conveniences care must be taken to protect them from rain, as their effectual operation to a large extent depends upon free access of air and constant evaporation. No better urinal, he says, can be devised than a tall bushel basket filled with deal sawdust and placed in a shed. Such an arrangement will in dry hot weather take a surprising amount of urine without affording any filtrate, owing to the freedom of evaporation; and if its use is not excessive, and if it be occasionally stirred, it will actually increase in effectiveness with use, the sawdust becoming strongly ammoniacal but not offensive. This is probably due to the action of micro-organisms. Dr. Poore says that he recently examined some sawdust which had been used for three months in the urinal of a country public-house, and found it absolutely free from all offensive odour. Could not some of our railway companies make a trial of this simple plan in some of their country stations? Station urinals are generally fairly attended to in London, but in many country places their condition is too abominable for words. Generally this is entirely the result of carelessness, water being allowed to run to waste all day long without ever reaching the spot where it is wanted. Perhaps the provision of a basketful of sawdust would come more within the organising capacity of a country station master.

